# Innovative Lightweight Concrete with Carbonated Magnesium-Based Pellets

**DOI:** 10.3390/ma19051038

**Published:** 2026-03-09

**Authors:** Onur Sahin, Enis Coşkun, Abdullah Huzeyfe Akca

**Affiliations:** Department of Civil Engineering, Yildiz Technical University, Istanbul 34220, Türkiye; sahino@yildiz.edu.tr (O.S.); enis.coskun@std.yildiz.edu.tr (E.C.)

**Keywords:** magnesium oxide, artificial aggregates, compressive strength, carbonation

## Abstract

The construction industry requires sustainable building materials to reduce its environmental impact. While using these materials in newly constructed structures primarily focuses on environmental benefits, their application in the protection of architectural heritage presents an additional requirement. These materials must be physically and chemically compatible with historical substrates to ensure the longevity of the structure. Therefore, developing eco-friendly and compatible restoration materials is a significant concern. This study aims to produce artificial aggregates to develop lightweight concrete for structural interventions and reduce natural resource consumption (i.e., minimizing the destructive extraction of natural river sand and crushed stone aggregates). Magnesium-based binders were used to mimic the carbonation process of historical lime mortars. The binders were mixed with water, shaped into coarse pellets, and cured in a ${CO}_{2}$ incubator for 3 and 14 days before being used in concrete production. The results show that using artificial aggregates decreased the concrete density by approximately 16.5%. Since reducing the dead load improves the seismic safety of historical masonry structures, this reduction is critical. Although the compressive strength decreased compared to natural aggregate concrete, the 14-day cured series achieved a strength of 34.7 MPa. This demonstrates that the material can be used in restoration interventions where stiffness compatibility is essential (e.g., vault infills, ring beams, or floor screeds). At the same time, since magnesium-based artificial lightweight pellets have ${CO}_{2}$ sequestration capacity, they can be used as a carbon-negative solution for both historical structures and broader civil infrastructure.

## 1. Introduction

Global construction activities consume natural resources, especially natural aggregates, at an alarming rate, and the construction sector is responsible for greenhouse gas emissions due to the energy-intensive production of Portland cement [1,2,3,4]. Therefore, the transition toward sustainable infrastructure is critical [5,6]. However, the development and the adoption of green materials can pose significant economic and technical challenges [7,8,9]. In order to make an environmentally friendly alternative a preferred construction material, it must offer more than just environmental benefits; it also requires superior functional characteristics such as improved strength-to-weight ratios, durability, or carbon sequestration capabilities.

Considering the seismic safety of the buildings and the sustainability of natural resources, artificial lightweight aggregates can be considered as a practical solution. For example, industrial by-products like fly ash can be transformed into artificial pellet aggregates [10,11,12,13]. The final product, referred to as cold-bonded pellets, addresses waste management while simultaneously reducing structural dead loads [14,15]. This reduction in dead load can also be vital for the preservation of historical buildings. Unlike modern structures, which are designed according to contemporary seismic codes with inherent flexibility and energy dissipation capacities, traditional masonry structures are significantly more susceptible to damage from seismic forces due to their high mass and lack of ductility. Therefore, replacing heavy infill materials with lightweight and compatible alternatives during restoration, such as in vault filling applications or floor leveling works, can mitigate the effects of seismic forces. Furthermore, artificial lightweight aggregates can positively influence the thermal properties of concrete [16,17,18].

Moving beyond simple waste utilization, recent trends in material science have shifted toward Carbon Capture and Utilization (CCU) technologies, seeking materials that actively remediate the environment, given that Portland cement production is responsible for approximately 8% of anthropogenic ${CO}_{2}$ emissions [19]. One of the most promising candidates for an environmentally friendly alternative to conventional calcium based-cement is magnesium-based cement. However, the environmental footprint of magnesium cement is source-dependent. Magnesium oxide (MgO) derived from magnesite $\left({MgCO}_{3}\right)$ often carries a carbon footprint comparable to or exceeding that of Portland cement due to the high temperatures required for calcination [20,21]. On the other hand, magnesium hydroxide $\left({Mg\left(OH\right)}_{2}\right)$ presents a fundamentally different opportunity which helps to create carbon negative construction materials. Unlike hydraulic binders, ${Mg(OH)}_{2}$ hardens via a carbonation mechanism, effectively locking ${CO}_{2}$ into the matrix by transforming it into stable magnesium carbonates [22]. To maximize these environmental benefits and ensure large-scale economic feasibility, these magnesium precursors can be abundantly sourced from industrial waste streams, such as desalination reject brines and low-grade magnesite mining tailings [23,24]. For instance, the global production of desalination brine could potentially supply approximately 172 million tons of MgO annually [25]. At the same time, synthetic magnesium precipitates derived from such brines can readily be processed into fine powders, e.g., finer than 150 µm [26]. Since the utilization of these materials as a binder requires fine particle sizes for effective reactivity, these specific dimensions and massive quantities make them highly compatible for various large-scale structural applications. Utilizing such by-products transforms these binders into fully sustainable, carbon-negative alternatives by simultaneously valorizing massive waste stockpiles and sequestering ${CO}_{2}$.

The hardening mechanism of magnesium oxide and magnesium hydroxide relies on the formation of hydrated magnesium carbonates (HMC), which bind particles together. The most frequently observed formations of HMC are nesquehonite, hydromagnesite, and dypingite phases (Equations (1)–(3)) [9]. However, these products emerge at the surface of the material first and densify the outer layer of the material, causing a reduction in the penetration ability of carbon dioxide [27]. As specimen dimensions decrease, the ratio of carbonated volume compared to the total volume of the material will increase. Unlike large concrete blocks, the small dimensions of these pellets facilitate rapid and uniform ${CO}_{2}$ ingress, potentially accelerating the curing cycle and ensuring homogenous carbonation products. From a conservation perspective, the resulting magnesium carbonates share physicochemical similarities with the calcium carbonates found in historical lime mortars. Furthermore, the carbon dioxide curing process essentially acts as an accelerated simulation of the natural aging process of historical materials. Therefore, pre-carbonated aggregates are less likely to cause deleterious reactions or volume changes when integrated into heritage structures.

(1) $${Mg(OH)}_{2}+{CO}_{2}+2H_{2}O\to {MgCO}_{3}\cdot 3H_{2}O\;\mathrm{(Nesquehonite)}$$

(2) $$5{MgOH}_{2}+4{CO}_{2}\to 4{MgCO}_{3}\cdot 4{Mg(OH)}_{2}\cdot 4H_{2}O\;\mathrm{(Hydromagnesite)}$$

(3) $$5{MgOH}_{2}+4{CO}_{2}+H_{2}O\to 4{MgCO}_{3}\cdot {Mg(OH)}_{2}\cdot 5H_{2}O\;\mathrm{(Dypingite)}$$

Accordingly, this study focuses on the production of artificial magnesium-based lightweight coarse pellets via a cold-bonding process followed by carbonation curing. The objective is to demonstrate the feasibility of a lightweight concrete that incorporates carbonated magnesium pellets as aggregates, offering a dual benefit: maximizing permanent ${CO}_{2}$ storage within the built environment and providing a lightweight, chemically compatible material solution for the sustainable restoration of architectural heritage.

## 2. Materials and Methods

In this study, commercial magnesium oxide and magnesium hydroxide powders were utilized to produce magnesium-based artificial pellets. The pure materials were used to establish a controlled baseline for microstructural analyses and precisely investigate the carbonation mechanisms without the interference of impurities. Concentrated carbon dioxide curing was applied to artificial pellets for strength development. Subsequently, these artificial pellets were incorporated into conventional Portland cement concrete as coarse aggregates. The physical and mechanical properties of both artificial aggregates and concrete specimens were determined.

### 2.1. Materials and Artificial Aggregate Production

MgO and ${Mg(OH)}_{2}$ powders were used as binders in pellet production and the maximum particle diameter of the powders was less than 150 μm. Additionally, natural sand was incorporated into specific pellet groups to increase the surface roughness of the particles. MgO powder had a specific gravity of 3.69 and a purity of 99.5% while ${Mg(OH)}_{2}$ powder had a purity range of 96–98% and a specific gravity of 2.36. The specific gravity of natural sand was 2.65.

**Figure 1:**
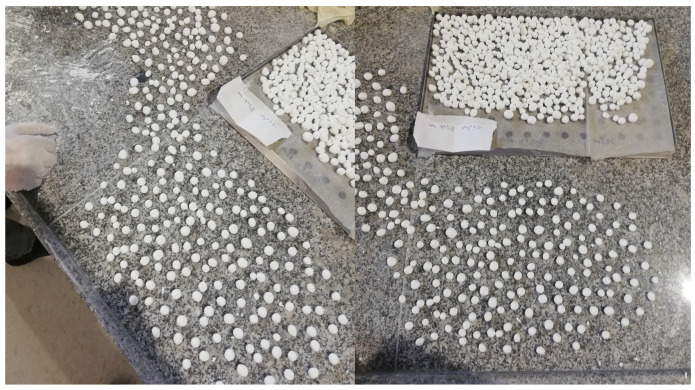
Magnesium-based pellet production.

Paste was obtained by mixing the dry powders with distilled water and the resulting mixture was manually shaped into spherical pellets (Figure 1). To systematically investigate the effects of different compositions on the carbonation efficiency and final aggregate properties, three distinct groups were designed: the ‘M’ series (pure MgO reference), the ‘MS’ series (incorporating silica sand as an inert micro-filler at a 1:2 binder-to-sand mass ratio to evaluate structural skeleton effects) and the ‘MH’ series (using a 1:1 mass ratio of MgO to ${Mg(OH)}_{2}$ to observe the influence of a pre-hydrated precursor).

The water-to-binder (W/B) ratio (where the binder is defined as the total mass of the reactive powders MgO and ${Mg(OH)}_{2}$) was determined experimentally for each group. Because the specific surface area and water demand of the mixtures varied significantly with the addition of sand or ${Mg(OH)}_{2}$, the W/B ratio was adjusted to maintain a consistent, moldable plasticity required for manual pelletization. Following pelletization, the aggregates were subjected to accelerated ${CO}_{2}$ curing for 3 and 14 days. These specific curing durations were selected to evaluate both the early-age rapid carbonation phase (3 days) and the advanced densification phase (14 days), enabling a comparative assessment of strength development over time. The detailed mixed proportions of the pellet aggregates are given in Table 1.

The target size range for the magnesium-based pellets (8–20 mm) was determined to replicate the standard fraction of natural coarse aggregates and to satisfy the specific dimensional requirements of the mechanical tests. According to the BS 812-111 standard [28], the Ten Percent Fines Value (TFV) test requires an aggregate fraction passing the 14.0 mm sieve and retained on the 10.0 mm sieve. Furthermore, reliable single-particle crushing tests in the literature typically require aggregate diameters larger than 10 mm to minimize size-effect errors and ensure accurate stress distribution. Following the manual pelletization process, which naturally yielded a distributed size range, the aggregates were sieved to isolate this targeted 8–20 mm functional fraction.

The pellets were exposed to carbon dioxide curing in a carbon dioxide incubator maintained at a carbon dioxide concentration of 10 ± 0.2%, temperature of 25 ± 3 °C, and relative humidity of 80 ± 5%. All of the groups were kept in their curing medium for durations of 3 and 14 days. Following the curing periods, mass change measurements, particle crushing strength tests, Ten Percent Fines Value (TFV) tests, X-Ray diffraction (XRD), and thermal gravimetric analyses (TGA) were conducted on the pellet aggregates.

#### 2.1.1. Mass Change Measurements

The initial mass of the pellets was recorded immediately after production, and the final mass of the pellets was measured at the end of their curing period. The percentage mass change was calculated based on the difference between the final and initial masses relative to the initial mass.

#### 2.1.2. Particle Crushing Test

**Figure 2:**
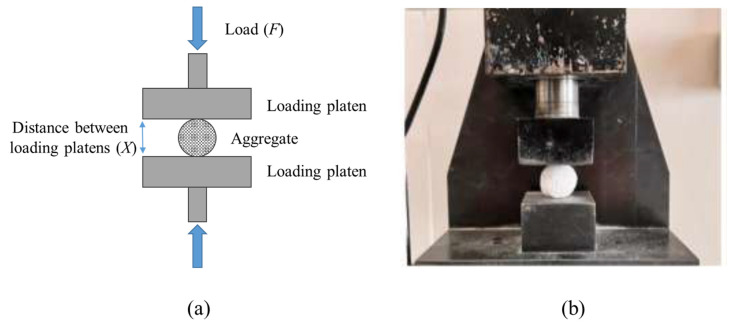
Aggregate crushing strength configuration (**a**) schematic, (**b**) during test.

Individual pellets were subjected to crushing loads between two parallel loading platens, as shown in Figure 2. Since the diameter of aggregates varied between 8 mm and 20 mm, the loading rate was adjusted in accordance with the diameter of the test sample so that the loading rate was 0.1 X/min, where X represents the initial distance between loading platens. Subsequently, the crushing strength of each aggregate particle was calculated by using Equation (4) [29,30].

(4) $$\sigma_{crushing}=\frac{2.8F}{\pi X^{2}}$$

The coefficient 2.8 in Equation (4) is derived from the theoretical stress distribution analysis established by Hiramatsu and Oka [31] for spherical and irregular particles compressed between two parallel flat platens. The aggregate diameters varied between 8 mm and 20 mm but Equation (4) inherently normalizes the failure load (*F*) by the square of the particle diameter (*X*^2^), yielding a size-independent stress value. In fact, this wide size range was deliberately selected to systematically investigate the effect of particle diameter on the ${CO}_{2}$ diffusion depth and carbonation efficiency, as evaluated in Section 3.

#### 2.1.3. Ten Percent Fines Value (TFV) Test

The crushing resistance of the aggregate samples was evaluated using the Ten Percent Fines Value (TFV) test, conducted in accordance with BS 812-111 [28]. This method determines the load required to produce 10% fines (particles passing a 2.36 mm sieve) relative to the total aggregate mass. Before testing, the aggregate samples were oven-dried at a temperature of 45 ± 5 °C. The dried material was then sieved to isolate the standard test fraction, consisting of aggregates passing the 14.0 mm sieve and retained on the 10.0 mm sieve. Approximately 3 kg of sample was prepared for each test run. The test specimen was placed into a standard steel cylinder with a diameter of 150 mm. To ensure uniform density, the aggregate was placed in three equal layers; each layer was subjected to 25 strokes using a standard tamping rod. After leveling the surface, a plunger was inserted, and the assembly was placed in a compression testing machine. A load was applied at a uniform rate to achieve a total penetration of approximately 20 mm over a duration of 10 min. The magnitude of the applied load was estimated to produce a percentage of fines falling within the 7.5 to 12.5 range by using Equation (5).

(5) $$TFV=\frac{14\times f}{m+4}$$

where *TFV* represents the Ten Percent Fines Value expressed in kilonewtons (kN), *f* is the maximum load applied to the test specimen (in kN), and *m* is the proportion of fines (particles passing the 2.36 mm sieve) produced at the maximum load *f*, expressed as a percentage.

#### 2.1.4. XRD and TGA Sample Preparation Procedure

The specimens were ground using a steel mortar and pestle. To obtain a fine powder suitable for microstructural analysis, the ground sample was sieved through a 150 μm sieve. The powder samples were utilized for TGA and XRD analyses. For TGA, a sample mass of approximately 17 ± 5 mg was used, and the temperature was ramped from ambient temperature to 1000 °C at a heating rate of 10 °C/min under a nitrogen flow of 60 mL/min. Representative samples from the same powder were used to characterize the crystalline phases of hydration and carbonation products. For XRD analysis, the diffraction patterns were collected between 5° and 80° with a step size of 0.04°, the anode material was Cu, and the intended wavelength was K-α (with generator settings 40 mA and 45 kV).

#### 2.1.5. Concrete Production and Compression Test

CEM I 42.5 R Portland cement, sourced from the Akçansa Büyükçekmece plant (Büyükçekmece, İstanbul, Türkiye), was used in this study. Laboratory characterization of the cement indicated a specific gravity of 3.12 and a Blaine specific surface area of 3740 cm^2^/g. The natural aggregate mixture was composed of three distinct fractions to ensure optimal particle packing. The coarse aggregate (No. I, 4–16 mm) and crushed sand (0–4 mm) were procured from the Güney Cebeci quarries (Sultangazi, İstanbul, Türkiye). The specific gravity values of the coarse aggregate and crushed sand were 2.70 and 2.69, respectively. Additionally, natural sand was incorporated into the mix design. This natural sand, supplied by Yılmazer Mining (extracted from the Kemerburgaz, Akpınar region, Şişli, İstanbul, Türkiye), exhibited a particle size distribution within the 0–4 mm range and a specific gravity of 2.60. To achieve the desired workability and performance, a polymer-based superplasticizer (Chryso Fluid Optima) was employed as the chemical admixture.

A total of 13 concrete mixtures were designed to investigate the effects of varying aggregate types and replacement ratios. The specific mix proportions and designations for each series are presented in Table 2. In these mixtures, artificial aggregates were utilized as a replacement for the natural coarse aggregate fraction. The mixtures consisted of 40% fine aggregate (20% crushed sand, 20% natural sand) and 60% coarse aggregate by volume. For the hybrid groups, a composite approach was adopted where artificial and natural coarse aggregates were blended at a 50:50 volumetric ratio.

For each mixture series, cubic specimens with dimensions of 100 mm cubic specimens were cast to evaluate the mechanical properties. The specimens were demolded 24 h after casting and subsequently cured in lime-saturated water until the testing ages of 7 and 28 days. Prior to the destructive compression tests, the water absorption of the samples was measured. Subsequently, three cubic specimens from each group were subjected to uniaxial compression at a constant loading rate of 0.6 MPa/s until failure. In accordance with standard testing procedures for cubic specimens (TS EN 12390-3) [32], the compressive load was applied perpendicularly to the direction of casting, directly onto the smooth, molded steel faces. The ultimate compressive strength values were determined based on the peak load recorded for each series.

## 3. Results

The experimental study was conducted in two distinct stages. In the initial phase, magnesium-based artificial pellets were produced and cured within a high-concentration carbon dioxide environment. Following the evaluation of their mechanical and microstructural properties, the second phase involved the production of concrete batches incorporating natural, artificial, and hybrid aggregate combinations. Finally, the performance of 13 different mixtures, including a reference control group, was assessed through water absorption and compressive strength tests on cubic specimens.

### 3.1. Mass Change Measurements After Carbon Dioxide Curing

**Figure 3:**
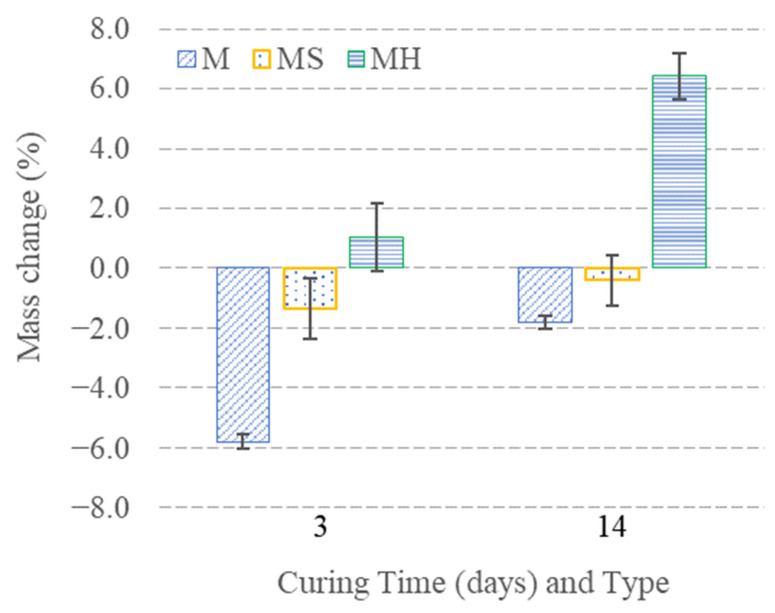
Mass change of the pellet groups.

Pellets were spread onto trays, and their initial mass was recorded prior to carbon dioxide curing. Subsequently, the trays were placed in the carbon dioxide incubator. At the end of the specified curing periods, the final mass of pellets was measured, and the net mass change was calculated (resulting from the combined effects of water loss and carbonation gain). The mass change results for different carbon dioxide curing periods are presented in Figure 3. The highest mass loss was observed in M3 and M14 groups, which also had the highest mixing water amount among all series. On the other hand, MH3 and MH14 groups showed a net mass increase during carbon dioxide curing. Regardless of the group, the final mass of all samples increased with extended curing time. The relative mass increases compared to day 3 were 4.0%, 1.0%, and 5.4% in M14, MS14, and MH14 groups, respectively. The mass gain is strongly correlated with the extent of carbonation of the pellets [33].

### 3.2. Physical Properties of Pellets

Following carbon dioxide curing, the pellets were dried at 45 °C for 24 h in an electric oven, and subsequently they were submerged in water for 24 h. The mass of the pellets was recorded in both dry and wet states. Based on these measurements, the oven dry (OD) particle density, saturated surface dry (SSD) particle density, and water absorption (by mass) were calculated, as presented in Table 3. The results indicate that all artificial pellet groups can be classified as lightweight aggregate in accordance with the TS EN 13055 standard [34]. The lowest particle density was obtained in M groups; this may be attributed to their high initial water content, which likely facilitated the formation of open pores within the microstructure. Consequently, the water absorption rate of the M3 group was the highest among all pellet groups. The highest particle density was observed in the MS14 group because the MS groups incorporate natural sand in their composition. Consistent with the mass change measurements, the density values of the pellet groups increased with curing time. The increases in oven-dry particle density were 7.6%, 4.1%, and 8.3% in M14, MS14, and MH14 groups, respectively, compared to their 3-day cured counterparts.

### 3.3. Aggregate Crushing Test Results

**Figure 4:**
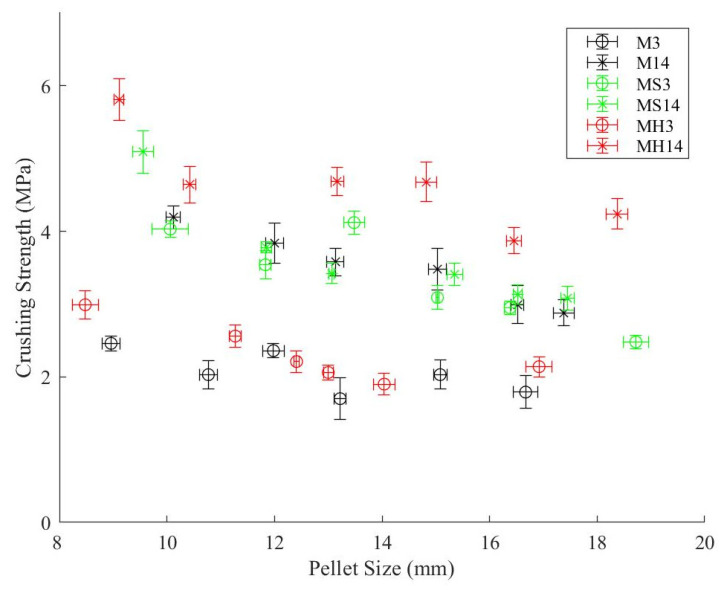
Relationship between single pellet crushing strength and particle diameter for different aggregate groups.

Pellet crushing tests were conducted on aggregates with diameters in the range of 8–20 mm, and the crushing loads were recorded. Subsequently, the crushing strength of each aggregate was calculated by using Equation (1). The relationship between the crushing strength and pellet size is illustrated in Figure 4. It should be noted that while the overall production range of the aggregates was 8–20 mm, the maximum average diameter plotted in Figure 4 terminates around 18.5 mm. This is due to the strict geometric requirements of the particle crushing test. During the fresh paste stage, larger pellets (approaching 20 mm) exhibited slight flattening due to their self-weight. Because Equation (4) assumes a spherical geometry for accurate stress distribution, any flattened or irregular pellets were excluded from the crushing test. Only highly spherical specimens were selected. Furthermore, each data point in Figure 4 represents the average of approximately 10 individual pellet tests, which naturally shifted the maximum average plotted diameter slightly below the 20 mm upper bound. The crushing test results showed that the pellets having a smaller diameter had higher crushing strength, and this may be attributed to the higher surface area to volume ratio characteristic of smaller particles. A higher surface area to volume ratio may facilitate the carbon dioxide absorption rate, enhancing carbonation, and result in higher crushing strength. Pellets subjected to carbon dioxide curing for 14 days showed generally better performance compared to those cured for only 3 days. The strength development of the MS groups was the lowest, while the highest crushing strength development was observed in the MH groups. While the average crushing strength value of pellets was around 2.5 MPa after 3 days of carbon dioxide curing, this value increased to 4.0 MPa after 14 days. This corresponds to approximately 60% improvement in the crushing strength of pellets obtained through extended carbon dioxide curing.

### 3.4. Ten Percent Fines Value (TFV) Test Results

The prepared pellet samples were tested under compression, and the mass of the particles smaller than 2.36 mm was measured. Using Equation (5), the TFV value of each group was determined, and the results are shown in Table 4. The lowest TFV values were obtained in MS groups; however, this is likely due to the release of the sand particles from MS pellets. These sand particles likely increased the total mass of particles smaller than 2.36 mm, irrespective of the binder strength. Consequently, the calculated TFV results for MS groups might be underestimated compared to their actual strength. While most pellet groups were classified as low-strength class, the TFV of the MH14 groups was determined as 120 kN. This value classifies it as moderate strength, indicating that M14 is suitable for use in the production of normal strength concrete. Consistent with previous findings, the TFV of each group increased with extended curing time. It should be noted that the TFV tests were conducted on the pellets with sizes between 10 mm and 14 mm, where the individual crushing strength was higher than that of the pellets having a larger particle diameter.

### 3.5. Microstructural Analyses Results

**Figure 5:**
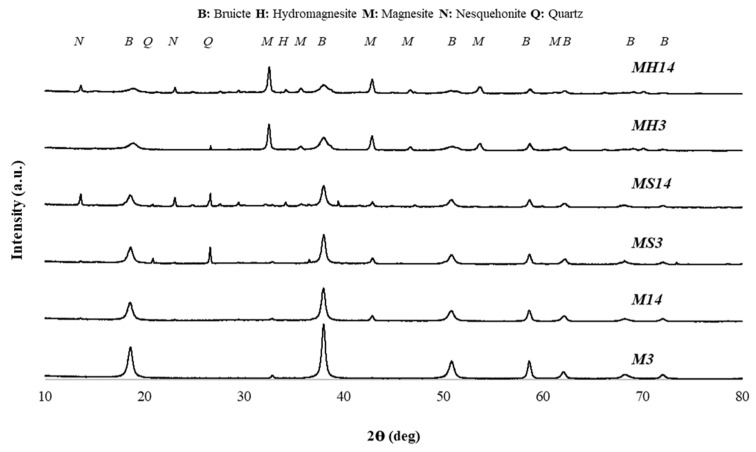
XRD patterns of pellets (Note: spectra are shifted vertically for clarity).

Pellets were ground in a ceramic mortar and pestle to obtain powder samples for XRD and TGA measurements. XRD analyses were conducted in a powder diffractometer using Cu Kα radiation (with generator settings 40 mA and 45 kV) to identify the crystalline phases, including residual binders and newly formed carbonation products. Diffraction patterns were collected between 5° and 80° with a step size of 0.04°. The XRD patterns of the pellets at different curing times are presented in Figure 5. Phase identification was performed using the ICDD Powder Diffraction Files (PDF).

Brucite was detected in all groups (magnesium hydroxide, PDF No. 44-1482) since magnesium oxide powder was the main binder of all the mixtures. This may indicate that after hydration of magnesium oxide, there are still uncarbonated magnesium hydroxide formations in the microstructure. The Quartz peaks (PDF No. 47-1144) detected in the MS groups are attributed to the presence of fine natural sand particles (<150 µm) that passed through the sieve during sample preparation. Peaks representing hydrated magnesium carbonates were detected in 14-day cured pellets (Nesquehonite, PDF No. 20-0669; Hydromagnesite, PDF No. 25-0513). On the other hand, magnesite peaks were observed in the XRD pattern of MH groups (Magnesite, PDF No. 08-0479).

**Figure 6:**
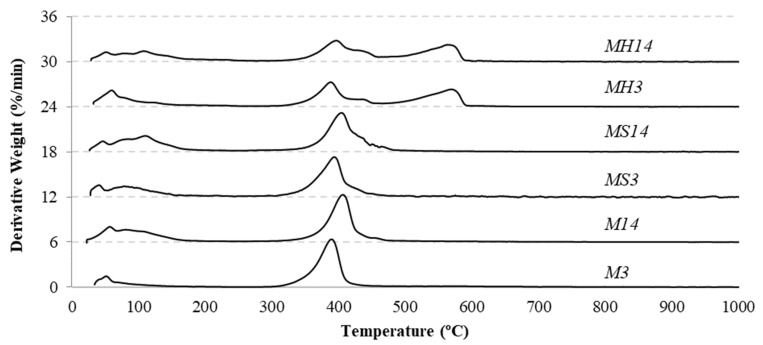
DTG curves of the pellets. (Note: For clarity of comparison, the curves are stacked, with each subsequent series shifted vertically by a constant offset of 6%/min).

Based on the TGA measurements, mass loss curves were plotted against temperature. From these, derivative thermogravimetry (DTG) curves were calculated to determine the rate of mass change, as shown in Figure 6. The peaks observed in the DTG profiles correspond to rapid mass loss events driven by the release of bound water and carbon dioxide. Assuming the residue remaining at 1000 °C consists purely of magnesium oxide [22], the final mass percentages of the TGA samples were recorded as 63.1%, 54.1%, 58.0%, 50.1%, 52.6% and 49.2% for the M3, M14, MS3, MS14, MH3, and MH14 groups, respectively. A lower magnesium oxide amount indicates that the sample contains more bound water and carbon dioxide in its structure.

In all the DTG patterns, a significant peak representing the dehydroxylation of magnesium hydroxide was observed at approximately 390 °C [35,36,37]. Although MH groups initially had magnesium hydroxide in their mixtures, the peak representing the dehydroxylation of magnesium hydroxide is lower than that of other groups, indicating that most of the magnesium hydroxide turned to carbonated forms after carbon dioxide curing. Nesquehonite decomposition initiates around 50 °C, the peaks below 250 °C correspond to hydromagnesite decomposition and the release of free water [6,38]. The peak adjacent to the magnesium hydroxide decomposition peak signifies the decarbonation of hydrated magnesium carbonates [39,40]. Additionally, the peak around 565 °C represents the decarbonation of magnesite [41].

By using the final mass, the mass loss due to dehydroxylation of magnesium hydroxide of the samples and the mass loss due to decarbonation of magnesium carbonate, the amount of carbon dioxide captured by the pellets can be estimated. The amount of magnesium hydroxide is calculated at first and then the remaining mass of the sample is determined. For M and MS groups the remaining formations can be considered as nesquehonite and hydromagnesite, for MH groups the remaining formations can be considered as nesquehonite, hydromagnesite and magnesite. By utilizing the stoichiometry of their molecular formulas, the amount of bonded carbon dioxide can be estimated. The results are presented in Table 5. For the MS groups, to account for the inert silica sand fraction, the calculated amount for each formation was multiplied by a factor of 2/3. Since determining the exact nesquehonite to hydromagnesite ratio was not feasible, an equimolar distribution (50:50 ratio) was assumed to simplify the calculations. The highest carbon dioxide absorption was obtained in the MH14 group, and it was around 28.5%. The calculations also showed that extending the curing time significantly enhanced the carbon dioxide sequestration of the pellets.

### 3.6. Test Results of Concrete Specimens

The mechanical performance and physical properties of the concrete specimens were evaluated through compressive strength tests and density measurements. For each mixture, three specimens were subjected to compressive strength testing, while a separate specimen was oven-dried at 45 °C for 7 days to determine the water absorption percentage. The specimens were weighed prior to the compressive strength testing and the results are presented in Table 6.

As expected, the lowest water absorption and the highest compressive strength and density were obtained in the reference control group. Conversely, the incorporation of artificial pellets resulted in a decrease in the density and the compressive strength of concrete, accompanied by an increase in water absorption. The use of magnesium oxide-based pellets reduced the density and the compressive strength of concrete by approximately 19.7% and 65.4%, respectively, compared to the reference concrete. Extended carbon dioxide curing enhanced the compressive strength of the concrete, albeit with a concurrent increase in density.

The hybrid concrete groups had higher compressive strength and density values compared to the groups containing 100% artificial pellets. Notably, the two highest compressive strength values among the lightweight mixtures were recorded in the MH hybrid series. Although the MS groups exhibited the lowest TFV results, their concrete compressive strength was higher than that of the M groups. This discrepancy suggests that the TFV test results may have been skewed by the release of loose sand particles, as previously discussed. Furthermore, the presence of sand particles on the pellet surface likely increased surface roughness, thereby enhancing the adhesion at the Interfacial Transition Zone (ITZ) between the aggregate and the cement paste. According to the TFV test results, the MH14 group was classified as moderate strength. And the compressive strength test results confirmed that the M14 group can be used in structures as normal strength concrete (Approximately C20/25).

## 4. Conclusions

Magnesium oxide, magnesium hydroxide and natural sand were utilized to produce artificial lightweight pellets using carbon dioxide curing. The pellets were cured in a carbon dioxide incubator maintaining 10% concentration for 3 and 14 days. After curing periods, the effects of carbonation were investigated via mechanical and microstructural tests conducted on artificial pellet aggregates. At the second stage of the study, the pellets were used as coarse aggregates in concrete mixtures and the influences of the pellet incorporation on the physical and the mechanical properties of concrete were evaluated.

The carbon dioxide exposure time can be considered as the most significant parameter effecting the properties of artificial aggregates. XRD patterns showed that each group had magnesium hydroxide in the microstructure and the amount of magnesium hydroxide was lower in 14-day cured pellets indicating higher amount of carbonation. Therefore, the peaks representing HMCs were also detected in the microstructure of pellets. The detection of magnesite peaks in the XRD patterns and the decomposition onset around 500 °C in TGA analysis indicate the synthesis of magnesium carbonate within the aggregate matrix of MH groups. The MH14 and MH14-H groups had superior mechanical properties compared to the other groups and it can be attributed to the formation of magnesium carbonate because magnesium carbonate may act as a strong binding agent. The reason for the increase in mass of the 14-day cured pellets should be the result of densification of the microstructure via extended carbonation and the magnesium based carbonates effectively filled the pores, enhancing the solidity of the pellets. This mechanism resembles the natural carbonation process of historical lime mortars but occurs within a very short period of time because of high concentration carbon dioxide curing. It should be noted that as long as magnesium hydroxide is present in the microstructure of pellet aggregates, strength development will continue through atmospheric carbon dioxide curing, ensuring a chemically stable material compatible with historical substrates.

The mechanical tests on concrete specimens produced with artificial lightweight pellets demonstrated a direct correlation with the curing duration. In both 100% and 50% coarse aggregate replacement ratios, the concrete groups containing 14-day cured aggregates exhibited, on average, 9% higher compressive strength compared to those with 3-day cured aggregates. The MH14 and MH14-H series achieved compressive strengths of 34.7 MPa and 42.0 MPa, respectively. These values not only meet the standards for normal-strength concrete, as predicted by the TFV results, but also exceed the typical strength requirements for restoration interventions in historical masonry. However, excessive strength can sometimes pose detrimental problems such as stiffness incompatibility. As a solution to this problem, the binder system can be modified by substituting Portland cement with calcium (CaO) or magnesium (MgO) based binders and this change allows the ultimate strength of the material to be tailored. Moreover, these binders adsorb carbon dioxide during their service of life and offer enhanced carbon dioxide sequestration capacity.

Furthermore, a significant reduction in density was observed in all concrete mixtures with the incorporation of artificial aggregates. In series where 100% of the coarse natural aggregate was replaced with artificial lightweight pellets, the concrete density decreased by an average of 16.5%, while the compressive strength decreased by 56.0%. For 50% replacement ratios, the reduction in density was 5.9%, with a 41.5% reduction in the compressive strength. Since the dry densities of the M3, MH3, and M14 groups were recorded below 2000 kg/m^3^ they can be classified as lightweight concrete. This density reduction has a critical importance for the seismic safety of historical structures. For example, by reducing the dead load of the intervention material (e.g., in vault infills or floor screeds), the overall seismic mass and base shear force can be minimized, offering a significant advantage over conventional normal-weight concrete repairs.

The environmental performance of the artificial pellet aggregates is as important as their mechanical performance. TGA measurements indicated that the MH14 aggregates chemically sequestered approximately 28.5% ${CO}_{2}$ by weight. When the total weight of the aggregates in a concrete mixture is considered, specifically at 100% coarse aggregate replacement, this corresponds to a permanent storage of approximately 225 kg of ${CO}_{2}$ per cubic meter of concrete. This carbon sequestration potential presents a dual benefit: mitigating carbon footprint of the construction sector and providing a functional alternative for the sustainable preservation of architectural heritage.

Although reducing the dead load is important for the seismic preservation of historical structures, carbon-negative lightweight aggregates also have potential applications in the transportation and civil infrastructure sectors. For instance, since these artificial aggregates have low density, they can be used as lightweight fill materials in highway embankments over soft soils to mitigate long-term settlement. At the same time, their porous nature makes them a promising material for pervious concrete pavements for sustainable stormwater management and the manufacturing of highway noise barriers. Moreover, when magnesium-based components are used in high-traffic environments, they can act as active carbon sinks. Since vehicle exhaust provides high localized ${CO}_{2}$ concentrations, the material can sustain carbonation over time. This continuous ${CO}_{2}$ curing can further densify the microstructure and increase the long-term mechanical strength.

This study focuses on the fundamental physical, microstructural, and compressive properties of the carbon-negative lightweight concrete. However, structural parameters such as shear strength and aggregate-matrix bonding performance were not evaluated. Since the artificial aggregates were produced manually using high-purity precursors to strictly observe the carbonation mechanisms, the production volume was limited. Conducting reliable macroscopic shear and bonding tests requires larger structural elements, which consumes a significant amount of aggregates. Therefore, future work will focus on scaling up the pelletization process using industrial waste streams, such as desalination brines or mining tailings. This will provide the necessary material volume to perform macro-structural investigations, including shear behavior, structural bonding, and flexural performance.

## Figures and Tables

**Table 1:** The mixed proportions of pellet aggregates.

Group	W/B Ratio	Water (kg/m^3^)	MgO (kg/m^3^)	${Mg(OH)}_{2}$ (kg/m^3^)	Silica Sand (kg/m^3^)	${CO}_{2}$ Curing Time (Days)
M3	1	787	787	0	0	3
M14	1	787	787	0	0	14
MS3	1.3	559	430	0	860	3
MS14	1.3	559	430	0	860	14
MH3	0.67	660	495	495	0	3
MH14	0.67	660	495	495	0	14

**Table 2:** Concrete mix proportions.

Groups	CEM I 42.5	Water	Artificial Pellet	Coarse Aggregate	Crushed Sand	Natural Sand	^1^ S.P. (1%)
	(kg/m^3^)						
Reference	360	160	0	1140	377	372	3.6
M3-H	360	160	358	570	377	372	3.6
M3	360	160	716	0	377	372	3.6
MS3-H	360	160	415	570	377	372	3.6
MS3	360	160	831	0	377	372	3.6
MH3-H	360	160	384	570	377	372	3.6
MH3	360	160	767	0	377	372	3.6
M14-H	360	160	367	570	377	372	3.6
M14	360	160	733	0	377	372	3.6
MS14-H	360	160	422	570	377	372	3.6
MS14	360	160	843	0	377	372	3.6
MH14-H	360	160	398	570	377	372	3.6
MH14	360	160	797	0	377	372	3.6

^1^ S.P. stands for superplasticizer (dosage represents 1% by weight of cement).

**Table 3:** Physical properties of the artificial pellets: Particle density and water absorption.

Group	OD Density (g/cm^3^)	SSD Density (g/cm^3^)	Water Absorption (%)
M3	1.30	1.69	30.3
M14	1.40	1.73	23.8
MS3	1.70	1.96	15.3
MS14	1.77	1.99	12.5
MH3	1.55	1.81	17.0
MH14	1.68	1.88	11.7

**Table 4:** Ten Percent Fines Value (TFV) test results and strength classification of the artificial pellets.

Group	TFV * (kN)	Evaluation **
M3	75	Low strength
M14	95	Low strength
MS3	60	Low strength
MS14	65	Low strength
MH3	90	Low strength
MH14	120	Moderate strength

* TFV values represent the result of a single test series for each group. In accordance with BS 812-111 [28], TFV results less than 100 kN are rounded to the nearest 5 kN, and results exceeding 100 kN are rounded to the nearest 10 kN. ** Strength classification: Low Strength (<100 kN), Moderate Strength (100–150 kN).

**Table 5:** Estimated mineralogical phase composition and ${CO}_{2}$ sequestration capacity of the artificial pellets (wt.%).

Phase	M3	M14	MS3 **	MS14 **	MH3	MH14
	(%)					
Brucite	77.8	68.1	51.9	47.6	41.6	37.3
Magnesite	-	-	-	-	32.1	38.4
Nesquehonite/ Hydromagnesite *	22.2	31.9	14.8	19.1	26.3	24.3
${CO}_{2}$ Uptake	7.7	11.0	5.1	6.6	25.9	28.5

* An equimolar distribution (50:50) was assumed for Nesquehonite and Hydromagnesite phases. ** For MS groups, calculations account for the inert silica sand fraction.

**Table 6:** Physical and mechanical properties of the concrete specimens at 28 days.

Groups	Dry Density (kg/m^3^)	Water Absorption (%)	Compressive Strength (MPa)
Reference	2399	1.2	68.0 ± 2.6
M3-H	2122	4.6	32.5 ± 1.6
M3	1928	5.5	23.5 ± 1.1
MS3-H	2273	2.6	32.6 ± 0.3
MS3	2087	4.1	30.9 ± 0.4
MH3-H	2253	2.5	45.4 ± 1.6
MH3	1982	4.0	31.8 ± 0.8
M14-H	2202	3.4	39.3 ± 1.6
M14	1997	5.0	28.5 ± 2.4
MS14-H	2260	2.8	39.9 ± 2.8
MS14	2107	3.5	30.0 ± 2.6
MH14-H	2297	2.6	42.0 ± 1.3
MH14	2065	4.5	34.7 ± 0.9

Note: -H represents hybrid mixtures containing 50% artificial and 50% natural coarse aggregates by volume. Density and water absorption measurements were conducted on a single specimen from each group. Compressive strength values represent the average of three specimens per group, accompanied by their standard deviations.

## Data Availability

The raw data supporting the conclusions of this article will be made available by the authors on request.

## Abbreviations

The following abbreviations are used in this manuscript:

a.u.	Arbitrary Unit
CCU	Carbon Capture and Utilization
DTG	Derivative Thermogravimetry
HMC	Hydrated Magnesium Carbonates
ICDD	International Centre for Diffraction Data
ITZ	Interfacial Transition Zone
OD	Oven Dry
PDF	Powder Diffraction File
SSD	Saturated Surface Dry
TFV	Ten Percent Fines Value
TGA	Thermal Gravimetric Analyses
W/B	Water-to-Binder
XRD	X-Ray Diffraction
